# Intraoperative perfusion assessment of the proximal colon by a visual grading system for safe anastomosis after resection in left-sided colorectal cancer patients

**DOI:** 10.1038/s41598-021-82486-9

**Published:** 2021-02-02

**Authors:** Hyo Seon Ryu, Seok-Byung Lim, Eu-Tteum Choi, Inho Song, Jong Lyul Lee, Chan Wook Kim, Yong Sik Yoon, In Ja Park, Chang Sik Yu, Jin Cheon Kim

**Affiliations:** 1 Division of Colon and Rectal Surgery, Department of Surgery, Asan Medical Center, University of Ulsan College of Medicine, 88 Olympic-ro 43-gil, Songpa-gu, Seoul, 05505 Korea; 2 Division of Nursing, Asan Medical Center, Seoul, Korea

**Keywords:** Anatomy, Gastroenterology, Signs and symptoms

## Abstract

We aimed to evaluate the clinical feasibility of a new visual grading system. We included 50 patients who underwent resection of primary colorectal cancer. Before anastomosis, the marginal vessel was cut and the perfusion status was assessed by a visual grading system. The visual grading system is comprised of five grades according to the bleeding from the marginal vessel and is categorized into 4 groups: good (grade A and B), moderate (grade C), poor (grade D) and none (grade E). Colorectal anastomosis was performed only in the good and moderate groups. We compared postoperative outcomes between the good and moderate groups and analysed the factors affecting the perfusion grade. Among the patients, 48% were grade A, 12% were grade B, and 40% were grade C. There was no anastomotic leakage. Only one patient with grade C showed ischemic colitis and needed reoperation. Age was the only factor correlated with perfusion grade in multivariate analysis (OR 1.080, 95% CI 1.006–1.159, *p* = 0.034). The perfusion grades were significantly different between > 65 and < 65 year-old patients (> 65, A 29.2% B 12.5% C 58.3% vs. < 65, A 65.4% B 11.5% C 23.1%, *p* = 0.006). Our intraoperative perfusion assessment that uses a cutting method and a visual grading system is simple and useful for performing a safe anastomosis after colorectal resection. If the perfusion grade is better than grade C, an anastomosis can be performed safely. Age was found to be an important factor affecting the perfusion grade.

## Introduction

An adequate blood supply is important for safe colorectal anastomosis in colorectal cancer surgery. Anastomotic healing defects, including anastomotic leakage (AL), are critical complications that may have a fatal impact on the patient after resection of colorectal cancer. In many cases, AL causes sepsis and requires percutaneous drainage or additional surgery. The occurrence of AL increases the length of hospital stay, the medical cost, and postoperative morbidity and mortality^1–3^. In addition, it can compromise oncologic outcomes^4^. In a recent meta-analysis, AL after restorative colorectal cancer surgery was associated with an increased incidence of local recurrence and reduced overall survival, cancer-specific survival, and disease-free survival^5^. The incidence of AL is reported to range from three to 11.6%^1–4^.

There is multifactorial aetiology in the development of AL, but an insufficient blood supply plays a substantial role^6,7^. For this reason, surgeons have tried to preserve a sufficient blood supply to the anastomotic area and assess the intestinal perfusion state more accurately before anastomosis. The most commonly used method for evaluating colonic perfusion is to observe the colour of the colon wall or the pulse of small vessels on the colonic mesentery^8^. This method is subjective and based on the operator’s experience, and may well lead to misinterpretations even by experienced surgeons. Other various tests using instruments, such as Doppler ultrasound^9^, laser Doppler flowmetry^10^, and oxygen spectroscopy^11^ have been applied to evaluate blood flow to the proximal colon. In recent years, the clinical utility of near-infrared fluorescence angiography with indocyanine green (ICG) has also been reported^12,13^. Notwithstanding, these tests other than the observation of the colour or pulse during surgery are not widely used due to the high price of the equipment, technical difficulties, and their lack of reproducibility^14^. Prolongation of the operation time due to the use of equipment can be a problem, and it is difficult to apply these tests or drugs in most institutions.

After the inferior mesenteric artery is ligated at its origin during left-sided colorectal cancer surgery, the blood supply of the bowel, which located in the proximal end, is provided by the marginal artery fed from the middle colic vessels^8^. We hypothesized that visual grading of the degree of blood supply in the marginal vessels on the proximal end of the anticipated anastomosis was possible, and a grading system could be utilized as an assessment tool for safe anastomosis after resection in left-sided colorectal cancer patients. This study aimed to evaluate the clinical feasibility of a new visual grading system for safe colorectal anastomosis and to identify clinical factors associated with the perfusion grade in left-sided colorectal cancer patients.

## Materials and methods

### Study population

We enrolled 50 consecutive patients with primary sigmoid colon or rectal cancer who underwent curative-intent surgery at the Asan Medical Center, Seoul, South Korea, between October 2019 and April 2020. The tumor location was classified into three groups: sigmoid colon, rectal cancer located above the peritoneal reflection (RA), and rectal cancer located below peritoneal reflection (RB). All operations were performed using standardized techniques by a qualified single surgeon (S-B Lim). The medical data, including the patient’s demographic findings, clinicopathological characteristics, operative findings including the visual grade of the marginal vessels, short-term outcomes, and morbidities were prospectively recorded in the database of our centre and then evaluated. This study was approved by the Institutional Review Board of the Asan Medical Center (IRB No. 2020-0749). An informed consent was obtained from all participants and all methods were carried out in accordance with relevant guidelines and regulations.

### Operative procedure and intraoperative perfusion assessment by the new visual grading system

All of the patients underwent a mechanical bowel preparation with citric acid hydrate/magnesium oxide/sodium picosulfate and skin hair removal by cream the day before the operation. As antibiotic prophylaxis, second-generation cephalosporin was administered 30 min before skin incision. A conventional laparoscopic approach was preferred, and an open approach was used for patients with a past medical history of open abdominal surgery.

After surgical exploration, the inferior mesenteric artery was ligated at the origin from the aorta (high ligation), and the inferior mesenteric vein was ligated at the level of the inferior border of the pancreas. In rectal cancer patients, a tumor-specific mesorectal excision was performed. In order to prevent tension on the colorectal anastomosis, to secure the proper length of the proximal colon, splenic flexure mobilization was performed if necessary. The distal colorectum was transected by a linear stapler with an adequate distal margin. After distal transection, the specimen including the tumor was extracted from the operation field with adequate wound protection.

When the proximal transection site had been established, the mesocolic fat, including the marginal vessel, was cut by a surgical scissor (not by an electrocautery probe) at the level of the planned anastomosis to assess the perfusion status of the proximal colonic end. One experienced surgical assistant (E-T Choi) assessed the perfusion status of the marginal vessels by applying the visual grading system predefined by the authors (Table 1). Anastomosis was performed when the grade was C (Moderate) or higher (grades A and B, Good). In cases of grade D (Poor) or E (None), the resection line for the planned anastomosis needed to be changed. The proximal intestine was resected until grade C or higher was confirmed.

**Table 1:** Visual grading system to assess the marginal vessel status.

Grade	Definition	Degree of perfusion
A	Brisk, bright red, ≥ 1 cm projectile bleeding	Good
B	Bright red, pulsatile, but not projectile bleeding	
C	Two colour bleeding with bright red arterial and the dark red venous blood, not pulsatile	Moderate
D	Only dark red venous blood is observed	Poor
E	No bleeding	None

The patients were divided into two groups according to their visual grade: Good (grade A and B) versus Moderate (grade C), and the variables between the two groups were compared. The patient’s vital signs, including diastolic blood pressure (DBP) and systolic blood pressure (SBP), were recorded from the anaesthetic monitoring equipment just as we cut the marginal vessel.

The anastomoses were constructed using a double-stapled technique. The inner diameter of the circular stapler was 28 or 29 mm depending on the bowel size of the patient. Reinforcement sutures were performed routinely except when the anastomosis level was too low to suture. In all cases, an air leak test showed no air leakage from the anastomosis site. A closed suction drain was inserted around the anastomosis area. The transanal tube was not used. Antibiotics were discontinued after one postoperative dose. Low molecular weight heparin was administered postoperatively and repeated once a day to the 5th postoperative day to prevent coagulation complications. In cases of anterior resection, sips of water were initiated on postoperative day 1, and on the 2nd day, patients started to eat a liquid diet and a soft diet on the 3rd day. For the patients who underwent a low anterior resection, we kept them on sips of water for two postoperative days, a liquid diet started on the 3rd day, and a soft diet on the 4th day. The drain was removed when there was no problem after eating a soft diet. The patients were discharged on the day after drain removal.

### Definition of postoperative morbidities

AL is defined as a defect of the intestinal wall integrity at the colorectal or colo-anal anastomotic site (including the suture and staple lines of the neorectal reservoir), leading to communication between the intra-and extraluminal compartments and a pelvic abscess close to the anastomosis^15^. Ischemic colitis was confirmed by abdominopelvic-computed tomography for patients showing suspicious clinical symptoms such as fever, persistent low abdominal pain, and tenderness. The CT showed decreased enhancement of the bowel segment proximal to the anastomosis. Postoperative ileus (POI) was defined in cases with at least two of the following five items occurring within the 30th postoperative day: nausea or vomiting over the preceding 12 h, inability to tolerate a solid or semisolid oral diet over the preceding two mealtimes, abdominal distension, no flatus or defecation over the preceding 24 h, and radiological evidence of postoperative ileus on an abdominal plain film or CT scan over the preceding 24 h^16^. A delayed diet was defined as the planned diet was delayed at more than three mealtimes.

### Statistical analysis

All continuous variables are given as mean with standard deviation (SD), and categorical variables are given as number and frequency. Clinicopathologic variables between the two groups (Good vs. Moderate) were compared using Chi-square or Fisher’s exact test as appropriate. Variables with *p* values < 0.05 on univariate analysis were selected for inclusion in the multivariate analysis. Multivariate analyses were conducted using log regression analysis to analyse odd ratios from which 95% confidence intervals were obtained. Statistical significance was established with a 2-sided test at *p* < 0.05. All statistical analyses were performed using IBM SPSS ver. 25.0 (IBM, Armonk, NY, USA).

## Results

### Clinicopathological characteristics and perfusion assessment

The clinicopathological characteristics of the patients are summarized in Table 2. A total of 50 consecutive patients with sigmoid colon cancer (n = 28, 56%) or rectal cancer (n = 22, 44%, RA 12 and RB 10) were included. The details of the surgery and perfusion assessment are shown in Table 3. When the mesorectum was cut and the perfusion was evaluated, the mean of systolic blood pressure (SBP) and diastolic blood pressure (DBP) at cutting marginal vessels were 106.3 ± 13.9 mmHg and 60.6 ± 8.4 mmHg, respectively. We found that 24 (48%) of these subjects had grade A, six (12%) had grade B, and 20 (40%) had grade C. Two patients had grade D when the first mesocolon was cut. One of these two was identified as grade B and the other as grade C once the section line was changed to a proximal site.

**Table 2:** Clinicopathological characteristics of the patients.

Variables	
**Age (years, mean ± SD**)	63.3 ± 11.8
**Sex (n, %)**	
Male	36 (72%)
Female	14 (28%)
**Body mass index (kg/m**^**2**^**, mean ± SD)**	24.3 ± 2.4
**Comorbidities (n, %)**	
Diabetes mellitus	18 (36%)
Hypertension	10 (20%)
Cerebrovascular disease	2 (4%)
Ischemic heart disease	1 (2%)
Atrial fibrillation	1 (2%)
Chronic kidney disease	1 (2%)
Liver cirrhosis	1 (2%)
**ASA score (n, %)**	
1	26 (52%)
2	24 (48%)
**Smoking (n, %)**	
Yes	25 (50%)
No	25 (50%)
**Serum cholesterol (mg/dL, mean ± SD)**	175.0 ± 41.0
**Preoperative chemoradiotherapy (n, %)**	
Yes	7 (14%)
No	43 (86%)
**Tumor location (n, %)**	
Sigmoid colon	28 (56%)
RA (above peritoneal reflection)	12 (24%)
RB (below peritoneal reflection)	10 (20%)
**TNM stage (n, %)**	
T stage	
1–2	16 (32%)
3–4	34 (68%)
N stage	
0	36 (72%)
1–2	14 (28%)
M stage	
0	47 (94%)
1	2 (4%)

*SD* standard deviation.

**Table 3:** Description of surgical procedure and perfusion assessment (n = 50).

**Procedures (n, %)**			
Anterior resection		19 (38%)	
Low anterior resection		18 (36%)	
Lowest anterior resection		13 (26%)	
**Technique (n, %)**			
Open		8 (16%)	
Laparoscopy^a^		42 (84%)	
**Diverting stoma (n, %)**			
Yes		10 (20%)	
No		40 (80%)	
**Operative time (minute, mean ± SD)**		114.9 ± 37.4	
**Anastomosis (n, %)**			
Stapled colorectal		49 (98%)	
Manual coloanal		1 (2%)	
**Reinforcement sutures (n, %)**			
Yes		39 (78%)	
No		11 (22%)	
**Blood pressure at cutting marginal vessel (mmHg, mean ± SD)**			
Systolic BP		106.3 ± 13.9	
Diastolic BP		60.6 ± 8.4	
**Perfusion assessment by visual grading system (n, %)**		Initial	Final
Good	A	24 (48%)	24 (48%)
	B	5 (10%)	6 (12%)
Moderate	C	19 (38%)	20 (40%)
Poor	D	2 (4%)	0
None	E	0	0

*SD* standard deviation.

^a^One case, open conversion due to tumor invasion to uterus.

### Postoperative outcomes

Postoperative course and morbidities are presented in Table 4. The planned diet was delayed in 10 (20%) patients, and three patients (6%) had POI. Two of the patients with POI required adhesiolysis, and the other improved after Levin tube decompression. Patients who had only delayed their diet were fed when bowel motility was restored after fasting and ambulation. No patient in this series experienced clinical AL. Only one patient who underwent AR showed colonic ischemia proximal to the anastomosis. This patient showed segmental ischemia at just above the anastomosis site on computed tomographic scan and sigmoidoscopy performed at postoperative day 4, and underwent a second operation (redo LAR).

**Table 4:** Postoperative outcomes.

Outcomes	
Postoperative hospital stay (excluding the day of surgery) (days, mean ± SD)	5.8 ± 3.2
Gas out (postoperative days, mean ± SD)	2.2 ± 0.8
Defecation (postoperative days, mean ± SD)	3.7 ± 1.2
Delayed diet (n, %)	10 (20%)
**Complications (n, %)**	
Ischemic colitis	1 (2%)
Anastomotic leakage	0
Postoperative ileus	3 (6%)

### Associations between factors and postoperative outcome with perfusion grade

Associations between the patient’s factors and perfusion grade were evaluated (Table 5). The grade A and B group (Good) were compared with the grade C group (Moderate). In the Moderate category, the mean age was significantly older (69.6 vs. 59.1, *p* = 0.001), and the proportion of patients with diabetes was significantly higher (55% vs. 23.3%, *p* = 0.028) than those of the Good group. The DBP and mean BP at cutting marginal vessels were significantly lower in the Moderate group (62.8 ± 7.9 vs. 57.3 ± 8.3, *p* = 0.020; 78.1 ± 8.8 vs. 72.5 ± 8.9, *p* = 0.034, respectively) than in the Good group. Age was confirmed to be associated with perfusion grade on multivariate analysis (OR 1.080; 95% CI 1.006–1.159; *p* = 0.034). ASA score, M stage, tumor location and procedure showed marginal significance between the two groups.

**Table 5:** Patient factors associated with perfusion according to grade (A + B vs. C).

	Grade A + B (n = 30)	Grade C (n = 20)	*p* value		OR	95% CI
			Univariate	Multivariate		
Sex (male)	20 (66.7%)	15 (75.0%)	0.538			
Age	59.1 ± 12.1	69.6 ± 8.2	0.001	0.034	1.080	1.006–1.159
BMI	24.6 ± 2.8	23.8 ± 1.8	0.307			
Smoking	16 (53.3%)	9 (45.0%)	0.573			
HTN	5 (16.7%)	5 (25.0%)	0.481			
DM	7 (23.3%)	11 (55.0%)	0.028	0.290	2.190	0.512–9.363
ASA score			0.051			
1	19 (63.3%)	7 (35.0%)				
2	11 (36.7%)	13 (65.0%)				
Cholesterol	181.9 ± 42.6	164.9 ± 37.3	0.154			
Cutting SBP	108.5 ± 14.5	103.0 ± 12.5	0.167			
Cutting DBP	62.8 ± 7.9	57.3 ± 8.3	0.020	0.353	0.908	0.740–1.114
Cutting MBP	78.1 ± 8.8	72.5 ± 8.9	0.034	0.799	1.023	0.857–1.222
PCRT	3 (10.0%)	4 (20.0%)	0.328			
TNM stage						
T stage			0.809			
1–2	10 (33.3%)	6 (30.0%)				
3–4	20 (66.7%)	14 (70.0%)				
N stage			0.098			
0	19 (63.3%)	17 (85%)				
1–2	11 (36.7%)	3 (15.0%)				
M stage			0.067			
0	28 (93.3%)	20 (100%)				
1	2 (6.7%)	0				
Tumor location			0.060			
SC	20 (66.7%)	8 (40.0%)				
RA	6 (20.0%)	6 (30.0%)				
RB	4 (13.3%)	6 (30.0%)				
Procedure			0.080			
AR	14 (46.7%)	5 (25.0%)				
LAR	11 (36.7%)	7 (35.0%)				
uLAR	5 (16.7%)	7 (35.0%)				

*OR* odds ratios, *CI* confidential interval, *BMI* body mass index, *HTN* hypertension, *DM* diabetes mellitus, *SBP* Systolic blood pressure, *DBP* diastolic blood pressure, *MBP* mean blood pressure, *PCRT* preoperative chemoradiotherapy, *SC* Sigmoid colon, *RA* reflection above, *RB* reflection below, *AR* anterior resection, *LAR* low anterior resection, *uLAR* lowest anterior resection.

A comparison of the postoperative outcomes between the Good and Moderate groups is shown in Table 6. In the Moderate group, the length of postoperative hospital stay was longer (6.8 ± 3.6 vs. 5.2 ± 2.7, *p* = 0.082) and gas out tended to be late (2.5 ± 0.8 vs. 2.0 ± 0.8, *p* = 0.074), but statistical significance was not achieved. Other variables were similar between the two groups.

**Table 6:** Postoperative outcomes associated with perfusion according to grade (A + B vs. C).

	Grade A + B (n = 30)	Grade C (n = 20)	*p* value
Postoperative hospital stay	5.2 ± 2.7	6.8 ± 3.6	0.082
Op time	111.0 ± 37.6	120.3 ± 37.4	0.418
Diet delaying	5 (16.7%)	5 (25%)	0.481
Gas out	2.0 ± 0.8	2.5 ± 0.8	0.074
Defecation	3.8 ± 1.2	3.6 ± 1.3	0.424
Complications	2 (6.6%)	2 (10.0%)	0.434
Ischemic colitis	0	1 (5%)	
Anastomotic leakage	0	0	
Postoperative ileus	2 (6.67%)	1 (5%)	

### Influence of age on perfusion grade and postoperative outcomes

The patients were divided into two groups according to their age (< 65 vs. ≥ 65), and the clinicopathologic characteristics and postoperative outcomes of the two age groups were compared (Table 7). In the older than 65 age group, the proportion of Moderate category (58.3% vs. 23.1%, *p* = 0.006) and the proportions of DM, HTN, and ASA grade 2 were significantly higher (DM: 54.2% vs. 19.2%, *p* = 0.010; HTN: 37.5% vs. 3.8%, *p* = 0.004, ASA grade 2: 66.7% vs. 30.8%, *p* = 0.010, respectively). Although there was no statistical significance, there was a tendency to have a slow recovery of bowel motility in patients over 65 years old (2.4 ± 1.0 vs. 2.0 ± 0.6, *p* = 0.076).

**Table 7:** Clinicopathological characteristics, distribution of perfusion grade and postoperative outcomes according to age.

	≤ 65 (n = 26)	> 65 (n = 24)		*p* value
Sex (male)	18 (69.2%)	17 (70.8%)		0.904
BMI	24.15 ± 2.6	24.39 ± 2.28		0.740
DM	5 (19.2%)	13 (54.2%)		0.010
HTN	1 (3.8%)	9 (37.5%)		0.004
ASA score				0.010
1	18 (69.2%)	8 (33.3%)		
2	8 (30.8%)	16 (66.7%)		
Smoking	13 (50%)	13 (50%)		> 0.999
Total cholesterol	195.1 ± 37.6	152.2 ± 32.3		< 0.001
Cutting SBP	107.9 ± 11.9	104.5 ± 15.8		0.399
Cutting DBP	62.6 ± 9.0	58.2 ± 7.3		0.078
Cutting MBP	77.8 ± 9.2	73.8 ± 8.9		0.134
PCRT				0.270
Yes	5 (19.2%)	2 (8.3%)		
No	21 (80.8%)	22 (91.7%)		
T stage				0.421
N stage				0.837
M stage				0.161
Tumor location				0.410
SC	14 (53.8%)	14(58.3%)		
RA	5 (19.2%)	7 (29.2%)		
RB	7 (26.9%)	3 (12.5)		
Grade				0.006
A	17 (65.4%)	7 (29.2%)		
B	3 (11.5%)	3 (12.5%)		
C	6 (23.1%)	14 (58.3%)		
Hospital stay	7.5 ± 3.1	7.4 ± 1.8		0.905
Diet delaying	4 (15.4%)	6 (25%)		0.406
Gas out	2.0 ± 0.6	2.4 ± 1.0		0.076
Defecation	3.5 ± 0.9	4.0 ± 1.5		0.126
Complications				0.759
Ischemic colitis	0	1 (4.2%)		
Anastomotic leakage	0	0		
Postoperative ileus	3 (11.5%)	0		

*BMI* body mass index, *HTN* hypertension, *DM* diabetes mellitus, *SBP* systolic blood pressure, *DBP* diastolic blood pressure, *MBP* mean blood pressure, *PCRT* preoperative chemoradiotherapy, *SC* sigmoid colon, *RA* reflection above, *RB* reflection below, *AR* anterior resection, *LAR* low anterior resection, *uLAR* lowest anterior resection.

## Discussion

The perfusion assessment method with a visual grading system that was evaluated in the present study has shown two clinically important points. First, the method could contribute to the creation of a safe anastomosis. In the present study, anastomosis-related complications occurred in only one patient (1/50, 2%), which is a better result than a previous report from our institution of a leakage rate of 5.6% after anterior resection in patients with rectal cancer^4^. Our patient, who required additional surgery due to anastomotic ischemia, had undergone anterior resection with sigmoid colon cancer. He was a 68-year-old man and was perfusion grade C. At the time of reoperation, a small thrombus was observed in the end vessels that branched from the marginal artery at the proximal resection site where the anastomosis was segmentally ischemic. So, although the exact cause is unknown, ischemia might be caused by an acute circulation blockage from a small thrombus forming during surgery. In general, ischemia occurring in an anastomosis area may progress to AL, and it is difficult to avoid faecal diversion in many of these cases. However, we were able to diagnose the patient with ischemic colitis before the progression of the AL, so his bowel continuity could be restored.

Second, the method used for performing this visual grading system is simple and easily acceptable. It did not use any special equipment or drugs. The surgeon and operating team could assess the perfusion status immediately without any delay in operation time. There was no possible harm to the patient, and the amount of blood loss during the assessment was negligible. Traditionally, the way to make sure that the blood flow of the bowel is appropriate is to see whether it is viable and healthy and to palpate the pulse. However, it is difficult to visually identify blood vessels and palpate the pulse in obese patients. In addition, such an interpretation is subjective. That is why it is difficult to set a standard, and even experienced surgeons can misinterpret the results of a traditional assessment. In contrast, the visual grading system we suggested has the advantage of being objective in regard to the degree of perfusion. Although its reproducibility has not been verified, there was little inter-personal differences in grading between operating team members. This minimal difference might be associated with the simplicity of the grading system, such as projectile, pulsatile, two colour bleeding, one colour bleeding and no bleeding. This simplicity and the ease of quantification of the grading system would also help inexperienced surgeons.

The present study demonstrated a significant association between perfusion grade and age. In the same context, another study reported that splanchnic blood flow decreases with age^17^, and this is consistent with several studies that reported that age is a risk factor for AL^18^. In the present study, the mean age of the Good group (grade A and B) was 60 and that of the Moderate group (grade C) was 70. Therefore, we divided the patients into two groups according to a cut off age of 65, but this might cause arbitrary bias. Nevertheless, the comparison results (less than 65 vs. older 65) showed several significant differences between the two groups. A decreased perfusion grade in elderly patients may be due to the presence of various comorbidities that can affect the vascular system. In the older than 65 group, comorbidities such as DM and HTN were more frequent than in the younger group. ASA, cholesterol, and DBP also showed significant differences between the two groups. In groups over 65 years of age, their physical condition has decreased, and this might have affected the perfusion grade. Nevertheless, this weakness does not worsen their clinical course after surgery. Although gas out seems to be half a day late and discharge is delayed by a day, there was no difference in terms of postoperative course, including postoperative morbidities. In the present study, the inferior mesenteric artery was ligated at the root from the aorta (that is, a high tie) considering complete lymph node dissection from the oncologic viewpoint in all included patients. The safety of a high-tie in restoring colectomy compared to low-tie (preserving the left colic artery) was not an issue in the present study. However, in the older than 65 age group, the need for the adoption of a low-tie might be considered for a safe anastomosis in left sided colorectal cancer patients. This issue should be evaluated in further study.

There were some limitations to our study. The method assesses the degree of bleeding in marginal vessels and does not directly assess the blood supply of the proximal colonic mucosa, in which the anastomotic healing process occurs. The status of the marginal vessels does not directly reflect the status of the colonic mucosa. Nevertheless, because the marginal vessels are the blood vessels closest to the anastomosis, evaluating the marginal vessels could be the most clinically feasible approach. The experiment of Allison et al.^19^ found that collaterals between the vasa recta could occur both extramurally as well as intramurally and Fallouji et al.^20^ showed that the mucoso-submucosal area received about two thirds of the incoming blood flow and seromuscular area received about one third of the incoming blood flow by dynamic flowmetry. Secondly, this method evaluated only the blood supply of the proximal end and did not consider the blood supply of the distal end, which is also important for a safe anastomosis. In some cases, problems of anastomosis can occur due to an abnormality of blood vessels in the distal end, causing venous congestion^21^. However, in general, the distal rectal stump has its own vascularization sustained by the middle and inferior rectal arteries, branches of the internal iliac artery^22^ and the possibility of vascular insufficiency of the distal rectum is extremely low in clinical practice. In the study, one experienced surgical assistant who knew the clinical status of patient participated in assessment the perfusion status, and it might be associated with some biases of the results in this study. In addition, the present study only evaluated short-term outcomes postoperatively. If there is a problem with the blood supply, it is diagnosed as ischemic colitis or AL in the acute stage, and in the chronic stage, vascular insufficiency could cause an anastomotic stricture. Therefore, it is necessary to follow-up the included patients for a longer period to evaluate the adequacy of the grading system.

## Conclusion

In conclusion, our visual grading system is a useful tool for the assessment of perfusion status after resection in left-sided colorectal cancer patients. This grading system could help with safe anastomosis. We found age was a significant factor affecting the grade. Future studies with a larger number of patients and long-term follow up are required.
